# Probing Surface and Interfacial Tension of Ionic Liquids in Vacuum with the Pendant Drop and Sessile Drop Method

**DOI:** 10.3390/ijms232113158

**Published:** 2022-10-29

**Authors:** Ulrike Paap, Bernd Kreß, Hans-Peter Steinrück, Florian Maier

**Affiliations:** Lehrstuhl für Physikalische Chemie II, Friedrich-Alexander-Universität Erlangen-Nürnberg, Egerlandstr. 3, 91058 Erlangen, Germany

**Keywords:** ionic liquids (ILs), surface tension, interface tension, pendant drop, sessile drop, surface science, vacuum

## Abstract

We report on the surface and interface tension measurements of the two ionic liquids (ILs) [C_8_C_1_Im][PF_6_] and [m(PEG_n_)_2_Im]I (*n* = 2, 4, 6) in a surface science approach. The measurements were performed in a newly developed and unique experimental setup, which allows for surface tension (ST) measurements using the pendant drop method and for contact angle measurements using the sessile drop method under the well-defined conditions of a high vacuum (from 10^−7^ mbar). The setup also allows for in vacuum transfer to an ultrahigh vacuum system for surface preparation and analysis, such as in angle-resolved X-ray photoelectron spectroscopy. For [C_8_C_1_Im][PF_6_], we observe a linear decrease in the surface tension with increasing temperature. The ST measured under high vacuum is consistently found to be larger than under ambient conditions, which is attributed to the influence of water uptake in air by the IL. For [m(PEG_n_)_2_Im]I (*n* = 2, 4, 6), we observe a decrease in the ST with increasing polyethylene glycol chain length in a vacuum, similar to very recent observations under 1 bar Argon. This decrease is attributed to an increasing enrichment of the PEG chains at the surface. The ST data obtained under these ultraclean conditions are essential for a fundamental understanding of the relevant parameters determining ST on the microscopic level and can serve as a benchmark for theoretical calculations, such as molecular dynamic simulations. In addition to the ST measurements, proof-of-principle data are presented for sessile drop measurements in HV, and a detailed description and characterization of the new setup is provided.

## 1. Introduction

The macroscopic property surface tension (ST) *γ* of a liquid is the change in surface free energy per change in surface area. It plays an important role for many applications, particularly, when coating, impregnation or spraying processes are involved. In a microscopic picture, to increase the surface area, molecules in the bulk have to break the attractive interactions with their neighbours to be exposed to a different environment (vacuum, gas phase or another liquid phase). Notably, we refer to the term “surface tension” for the liquid–gas and the liquid–vacuum interface; in case of the liquid being in contact with a second dense phase, such as a solid or another liquid, we will use the term “interfacial tension” [1,2]. Despite the highly dynamic situation at the liquid surface, on average, the molecular moieties with the least attractive interactions in the bulk are preferentially exposed at this outer surface. This general behaviour was already stated by Irving Langmuir in his so-called “Langmuir principle” many decades ago, according to which the surface tension is represented by “the result of superposition over the molecule parts present at the outer surface” [3]. The more non-isotropic the molecules of the liquid, the more complicated the prediction or modelling of its surface tension is. This holds particularly true for ionic liquids (ILs), which are salts characterized by low melting points, often even liquid below room temperature, and negligible vapour pressure. They are typically made from highly asymmetric and weakly coordinating ions, and thus their properties can be tailored over a wide range, which make them interesting for many applications [4,5,6,7]. The composition of the IL’s surface layer is governed by a preferential orientation of the ions towards the gas (or vacuum) side in order to minimize the surface free energy, which is then directly reflected in the experimentally observable surface tension [8].

These considerations illustrate that the surface tension of a liquid depends on the chemical composition of the surface layer, and thus will be sensitive to contaminations or the gaseous environment. On the one hand, surface-active residual contaminations dissolved in the bulk will preferentially be located at the outer surface. On the other hand, the gas environment might contain additional gas atoms/molecules apart from the liquid’s vapour, which can adsorb at the surface (e.g., volatile hydrocarbons or water from the air) or even react with surface species. Both sources for surface contamination or modification will have an impact on the measured surface tension [9,10]. In order to obtain a fundamental understanding on the intrinsic surface tension of a liquid, which can, e.g., serve as a benchmark for theoretical calculations such as molecular dynamic simulations, it is thus necessary to be able to accurately determine the intrinsic surface tension under well-defined and ultraclean conditions, that is, at best, in a high or even ultra-high vacuum (UHV). As has been shown over the last two decades, UHV-based surface science methods can be well applied to study the surface properties of ILs due to their extremely low vapour pressures [11,12,13,14,15]. This enables one to characterize the surface composition and the molecular arrangement of cations and anions with high accuracy, which in turn sets the stage for studying the surface tension of well-defined and well-characterized clean surfaces. The obtained correlation between surface tension and microscopic structure at the surface can then also be used to tailor surface properties for specific experiments in fundamental science, e.g., by using liquid mixtures or for targeted applications in industry.

The ST *γ* of a liquid can be determined with different experimental methods, such as the capillary rise, the Du Noüy ring, the Wilhelmy plate, the spinning drop, the maximum bubble pressure and the pendant drop method [16,17]. All of these methods have already been successfully applied to study the ST of ILs under non-vacuum conditions [18]. The pendant drop (PD) method is one of the most popular standard techniques and is used in various gas atmospheres and under different pressure conditions. As will be shown in this work, it is also suitable to investigate the ST of ILs under vacuum conditions, since the exact shape of a pendant IL drop at the end of a vertically mounted capillary can be recorded through a vacuum-tight window. Moreover, the pendant IL drop can be subsequently placed onto a well-defined and clean solid surface (e.g., a single metal crystal surface previously cleaned by argon sputtering and thermal annealing in UHV) to determine the intrinsic interfacial tension by recording contact angles (CA) without breaking the clean vacuum conditions. For both the PD and CA measurements, the physico-chemical background is shortly summarized in the following.

The exact determination of the shape of the pendant drop is the most important factor for the accuracy of PD surface tension measurements [16,19,20,21]. In 1881, Worthington had already proposed that the surface tension of a liquid can be determined from the shape of a hanging drop, which is deformed by gravity [22,23,24]. Shortly thereafter, in 1883, Bashford and Adams published numerical tables for the approximate solutions of the axially symmetric Young–Laplace equation [25]. The Young–Laplace equation is a nonlinear partial differential equation describing the capillary pressure difference sustained across the interface between the liquid and a gas phase (or across the interface between the liquid and another immiscible liquid phase) due to the surface (or the interfacial) tension *γ*. This equation is still used today to derive *γ.* The shape of the drop is determined by a single dimensionless quantity *β = Δρ* *g R_0_^2^/γ* as a measure of the ratio of gravitational and interfacial forces. Therein, *Δρ* is the density difference between the liquid and the second phase to account for the drop buoyancy, *g* the acceleration due to gravity and *R_0_* the radius of the drop curvature at its apex [25]. In 1947, *β* was defined as the ‘Bond number’ by Merrington and Richardson [26]. The surface (interfacial) tension thus can be directly obtained from the density, gravitational force and drop size if the bond number can be accurately determined from the drop shape. This, however, proved to be challenging for some systems, particularly for drops strongly elongated due to a low surface tension with just *R_0_* as the characteristic drop shape parameter. In 1938, Andreas et al. demonstrated an improved approach to determine *γ* from the aspherical drop shape [27]: this approach is based on the value *S = d_s_ / d_e_*, which is the ratio of the maximum drop diameter, d_e_, and the drop diameter, d_s_, measured at a distance d_e_ from the apex (see Figure S1 of Supporting Information, SI). This ratio *S* is then compared with tables calculated experimentally by Andreas et al., which correlate the bond number and interfacial tension. These tables were further improved over time by numerical integration of the Young–Laplace equation [27,28]. Today, standard programs to calculate surface tension are based on the ADSA algorithm (axisymmetric drop shape analysis) developed by Neumann et al. [19,29,30,31,32,33].

As already stated above, the wetting behaviour of liquids on solid surfaces is closely related to the solid–liquid interfacial tension $\gamma^{SL}$. For interface tension measurements between a liquid and a solid, the contact angle (CA) of a sessile drop is commonly measured [34]. In 1805, Young proposed a force balance equation in the thermodynamic equilibrium for the relation between the interfacial tension $\gamma^{SL}$ and the contact angle: $\cos\theta =\frac{\gamma^{SG}-\gamma^{SL}}{\gamma^{LG}}$. Therein, θ is the contact angle between liquid and solid, and $\gamma^{SG}$ and $\gamma^{LG}$ are the solid-gas and liquid–gas surface tensions, respectively [34,35,36]. From this Young equation, the impact of any impurities being present at the solid or liquid surface as well as at the solid-liquid interface becomes obvious. Thus, to obtain the intrinsic interfacial tension between a well-defined solid surface and an ultraclean IL, sessile drop measurements under ultraclean vacuum conditions are also extremely helpful.

Here, we report on a novel experimental setup for high vacuum (HV) pendant drop (PD) and sessile drop (SD) measurements developed in our group. It can operate in the pressure range from 10^−7^ mbar up to approx. 1 bar. Moreover, it allows for sample transfers without breaking vacuum conditions between this setup and another UHV instrument, the so-called Dual Analyzer System for Surface Analysis, DASSA [37]. This transfer allows us, on the one hand, to prepare and characterize a solid surface in the DASSA by sputtering, annealing, low-energy electron diffraction (LEED) and angle-resolved X-ray photoelectron spectroscopy (ARXPS) for subsequent sessile drop measurements. On the other hand, the surface composition of the IL used in the pendant drop experiments can be subsequently determined by ARXPS for a direct correlation between surface composition/purity and surface tension values measured without risking additional contaminations by sample transport in air.

In the following, we first present the new PD/SD setup. After calibration with the well-known standard systems of clean water and clean benzene in air, the first temperature-dependent surface tension measurements under vacuum conditions for ILs will be shown. The studied ILs, [C_8_C_1_Im][PF_6_] and [m(PEG_n_)_2_Im]I (*n* = 2, 4, 6), are characterized by very different chain substituents in the cation and different anions. The derived surface tension values will be discussed in light of the composition at the outermost surface determined by ARXPS along the Langmuir’s principle [8]. In addition, we demonstrate for [C_8_C_1_Im][PF_6_] in contact with stainless-steel the influence of impurities (as deduced by XPS) on the measured contact angle with the SD method.

## 2. Setup

### 2.1. General Setup

**Figure 1:**
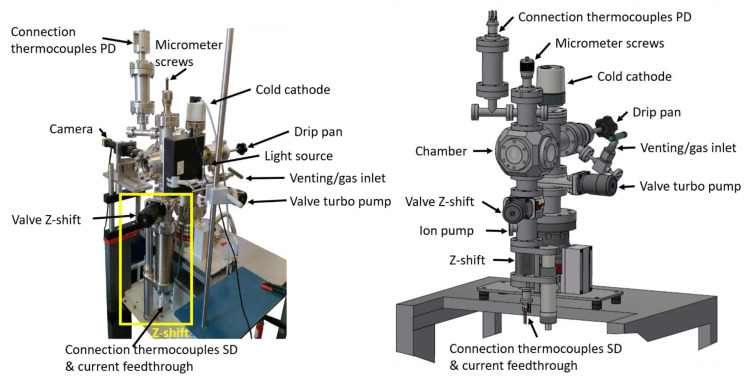
Photograph (left) and drawing (right) of the whole vacuum PD/SD system.

The new high vacuum (HV) pendant drop/sessile drop (PD/SD) apparatus operates in the pressure range from atmosphere (≈1 bar*)* to HV (10^−7^ mbar), and in a temperature range from room temperature to about 400 K (see Figure 1). The setup is mounted in a small stainless-steel vacuum six-way cubic cross as vacuum chamber, which is pumped by a turbomolecular pump (Pfeiffer, HiPace 80) combined with an oil-free scroll pump (Pfeiffer, HiScroll 6). In order to vary the pressure or introduce a certain gas atmosphere, the gate valve to the pumping is closed, and the chamber can be either vented with or gases can be introduced by a fine dosing valve. The pressure is recorded using a multi-range pirani/cold cathode gauge (Pfeiffer, PKR 251). Heating tapes allow for uniform baking the chamber, for degassing the liquids and for temperature-dependent measurements. Optical images of the pedant or sessile drops are recorded through a CF40 window using a high-speed camera (DataPhysics, iDS, UI-3350CP-M-GL R2); the drops are illuminated from the back by a diffuse LED light screen, mounted on the opposite window for accurate imaging [16,38]. In order to reduce reflections from the metallic walls inside the cross, a cover with minimum sized rectangular opening (10 × 25 mm^2^) is positioned at the illumination flange (that is, between the light source and the drop).

### 2.2. Pendant Drop

When performing pendant (and also sessile) drop measurements in vacuum, one has to consider specific boundary conditions. Most importantly, nearly all (conventional) liquids do simply evaporate at low pressures, which means they cannot withstand vacuum. Here, ILs are an exception, since most of them have a negligible vapour pressure at room temperature and thus are stable even under ultrahigh vacuum conditions. In addition, there is another very fundamental practical issue: In a standard PD/SD setup with a syringe operating under ambient pressure, the 1 bar pressure acting at the pendant drop surface allows for pushing out from or drawing back into the vertically mounted cannula by moving the syringe stamp, which enables an easy adjustment of a constant and stable drop volume. For measurements in vacuum, there is no pressure acting at the droplet surface, which implies that the liquid can only be pushed out while no negative pressure difference can be generated by pulling back the syringe stamp. The liquid droplet and the liquid inside the cannula is thus held back only by capillary forces (without capillary forces, it would simply leak from the syringe). Consequently, the cannula has to be designed such that for a drop of an appropriate size, the capillary forces are comparable to the gravitational ones (see also SI). To obtain suitable and relatively stable pendant drops for the ILs measured here, this is achieved by using a stainless-steel capillary of an outer and inner capillary diameter of 2.02 and 0.50 mm, respectively; in addition, two twisted stainless-steel wires (0.2 mm diameter) are inserted into the cannula in order to increase the contact area (and thus, the capillary forces) within the cannula (for more details, see Figure S2 of SI). When the drop size exceeds a certain size, the excessive gravitational forces eventually lead to the separation of the drop from the cannula.

**Figure 2:**
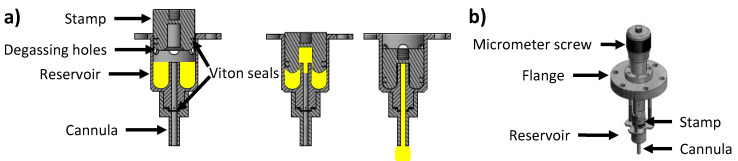
Concept and construction to form a drop in HV: (**a**) Representation of the inner part in the vacuum consisting of a stamp, reservoir with degassing holes and cannula. The stamp is pushed in the reservoir to press the liquid (in yellow) into the cannula forming a drop at its tip. (**b**) Full setup with the micrometre screw moving the stamp in a vertical direction.

In order to be able to perform PD measurements in a high vacuum, we had to build a specific new PD syringe setup. The self-designed construction made of stainless-steel consists of a stationary reservoir with holes for degassing, a replaceable cannula and a stamp that can be moved in vertical direction using a micrometre screw-drive (see Figure 2a). The whole setup is mounted on a CF 40 flange (see Figure 2b) such that the tip of the cannula will end in the centre of the six-way cross after mounting. Liquid tightness between the stamp and the reservoir, and between the cannula and the reservoir, is achieved via viton seals mounted on the stamp and between the cannula and the reservoir (see Figure 2a). The IL under investigation is filled into the reservoir in air with retracted stamp (that is, with open degassing holes), and the setup is then attached to the HV chamber via the top CF flange and carefully pumped down. Notably, great care has to be taken during initial pumping, since any traces of gases dissolved in the IL will lead to bubble formations in vacuum and, eventually, splashing of the IL inside the vacuum chamber. As detailed in the SI, the pumping and IL degassing take place over several hours at chamber temperatures of 70 up to 100 °C to remove any volatile species from the bulk liquid.

**Figure 3:**
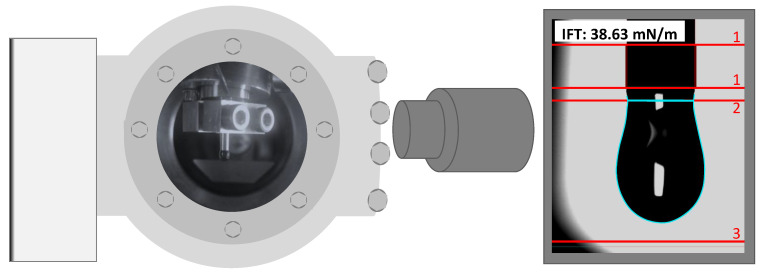
Left: Schematic representation of the PD setup with diffuse light source (left), chamber with drop (middle) and camera (right). Right: Drop image with manually set reference lines for surface tension evaluation (red, 1: magnification lines, 2: cannula end line, 3: boundary line for calibration) and obtained contour lines (turquoise).

Once the final vacuum is reached and the IL is fully degassed, the stamp is slowly moved down into the reservoir (thereby closing the degassing holes before touching the liquid surface), pushes the liquid into the cannula and, at the end of the cannula, a drop of growing size is slowly formed (see scheme of Figure 2a). Just before a targeted drop has reached its maximum size, that is, close to disconnection, the stamp must be moved back a little; otherwise, the IL is further pushed out of the reservoir into the cannula and the drop falls off very quickly. At this metastable stage, the pendant drop grows only very slowly due to gravity without significantly changing its shape. Eventually, it becomes too heavy and disconnects from the cannula and is collected in a glass vessel, which is placed under the cannula in an extra tube attached to the chamber. The challenge is to record the final shape of the drop just before it disconnects. While this is difficult when taking single photographs, it becomes easy when taking a video, where single images for PD analysis can be selected later. To illustrate the reproducibility for one single IL drop formed under these conditions, 100 ST values extracted from the final 20 s before drop disconnection (video rate: 5 frames/second) are shown in Figure S4 of the SI (for illustration of the growing drop size, four selected images are indicated; the full video is also provided in SI). Over the first 80 images, a very constant ST value of 43.47 ± 0.02 mN/m is deduced before a strong increase in the ST values starts at image 85, shortly before the drop is released. For the corresponding axisymmetric drop shape analysis (ADSA) of the drop shape and the self-consistent solution of the Young–Laplace equation, we used the SCA 22/15 software (surface and interfacial tension; pendant drop) from DataPhysics, which numerically solves the Young–Laplace equation in a self-consistent way with respect to manually set reference lines (see Figure 3).

**Figure 4:**
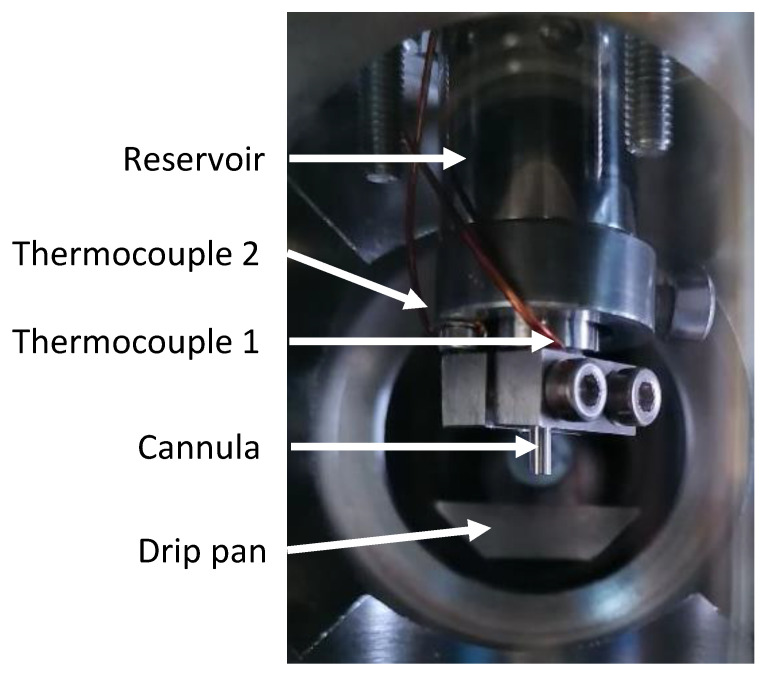
View into chamber for pendant drop measurements (note the two thermocouples attached to the cannula for accurate and independent temperature readings).

As mentioned above, the whole vacuum chamber is heated with heating tapes in order to determine surface tensions at different temperatures. As the mass and thermal inertia of the stainless-steel chamber is quite large and the heat conductance of the vacuum is zero, heating and cooling of a pendant drop is very slow, and it is very difficult to target a certain absolute and constant temperature. The videos for the temperature-dependent ST-measurements are, thus, typically recorded as close as possible to the targeted temperatures with as low a thermal drift of the cannula temperature as possible (around ± 0.1 K/min) to achieve good thermal equilibration. Note that the accuracy of the temperature determination at the location of the pendant drop is, nevertheless, very precise (± 0.2 K). The homogeneity of the temperature is verified by two independent type K-thermocouples, which are mounted next to the cannula tip (see Figure 4); the maximum difference between the two thermocouples is 0.2 K. Data at specific absolute temperatures values (e.g., the standard temperature of 298.15 K or other ST values of ILs given in literature) are obtained by linear interpolating our ST values measured for temperatures below and above the targeted temperature.

### 2.3. Sessile Drop

Our system can also be used for sessile drop measurements onto ultraclean surfaces. For these measurements, we use a cannula with a smaller outer diameter of Ø_o_ = 1.05 mm (inner diameter: Ø_i_ = 0.5 mm) in order to obtain a suitable small size of the formed droplets. According to literature [34,39,40,41,42,43], the volume of a sessile drop should be 0.5 to 10 μL so that the influence of the gravitational force can be neglected and reproducible data are obtained. After pumping down the chamber and degassing the liquid in the reservoir, first, a few preliminary drops are formed and disposed into a small drip pan, which can be moved under the cannula. Thereafter, the actual deposition of droplets on solid samples is performed.

The sample is mounted on a z-shift manipulator in a vacuum suitcase, which is attached to the bottom flange of the PD/SD chamber via a gate valve and is pumped by a small ion getter pump. The transport suitcase allows for the transfer of well-prepared samples (e.g., cleaning by sputtering and annealing in UHV, followed by surface analysis by XPS) via a fast entry load-lock (FEL) chamber from our UHV XPS system DASSA [37] to the PD/SD chamber without breaking the vacuum. Vice versa, it also allows for vacuum-transfer of deposited sessile drops without air contact to the DASSA for further XPS analysis with respect to IL composition and purity.

**Figure 5:**
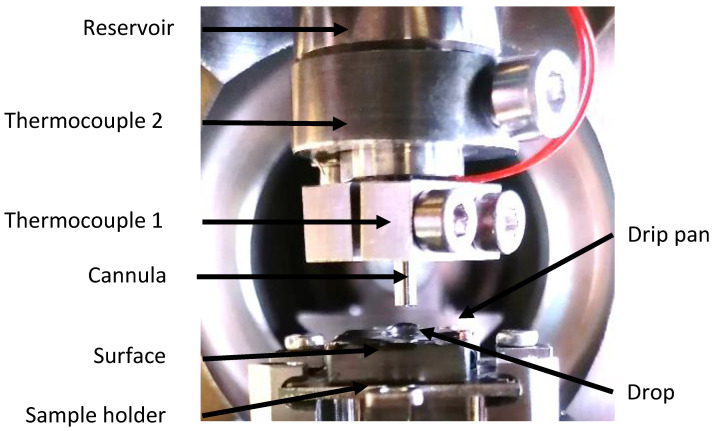
View into chamber for sessile drop measurements. A sample holder is positioned directly under the cannula to place a drop on a sample surface.

**Figure 6:**
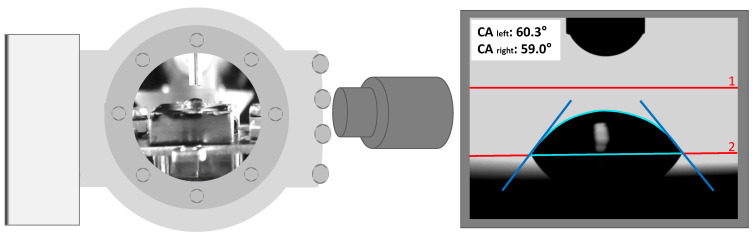
Left: Schematic representation of the SD configuration for interface tension measurements with the diffuse light source (left), chamber with sessile drop (middle) and camera (right). Right: Image of a sessile drop of [C_8_C_1_Im][PF_6_] placed at room temperature under vacuum conditions on carbon-contaminated stainless-steel with the manually set reference lines in red (1: boundary line, 2: base line). From the software-derived contour line (turquoise) with its tangents (blue), the values for the left and the right contact angle are deduced.

In order to deposit a drop onto the solid sample surface, the gate valve is opened and the sample is moved into the targeted position below the cannula with the z-shift (see Figure 1 and Figure 5). After deposition of the droplet, the contact angle is determined by taking photographs with the camera and analysing the images using the software SCA 20 (contact angle measurement) from DataPhysics (see Figure 6). A pBN heater (Neyco, PCPBNP05, Φ = 12.7 mm, resistance: 5–7 Ohm, maximum power: 80 W) mounted in the z-shift allows for simultaneous heating of the sample (up to 800 K) in order to perform temperature-dependent contact angle measurements.

In a preliminary test in air, contact angles (CA) of a drop of the IL [C_8_C_1_Im][PF_6_] (see next section also) on two different stainless-steel samples were measured (see photographs, CA values and ex situ survey XP spectra in Figure S5 in SI). The sample surface with a larger carbon content in XPS (and an additional fluorine contamination) exhibited a considerably larger CA (~54°) and, thus, a worse IL wetting behaviour compared to the the less-carbon contaminated surface, where a significantly smaller CA (~24°) was measured. These first findings are in line with our earlier studies on the change in IL wetting behaviour with surface carbon content in cases of ultrathin IL films deposited by physical vapour deposition onto various carbonaceous surfaces [44]. The proof-of-principle sessile drop experiment under vacuum is shown in Figure 6: a small drop of [C_8_C_1_Im][PF_6_] was placed under HV conditions on a stainless-steel sample holder confirming that well-defined sessile drops for reliable CA measurements in vacuum can be performed with our setup. In the future, detailed SD measurements of clean ILs in contact with well-defined single crystal surfaces are foreseen.

## 3. Reference Pendant Drop Measurements in Air

When introducing a new experimental apparatus, it is essential to perform calibration measurements under well-defined conditions. For this purpose, we decided to measure the surface tensions of water and benzene under ambient conditions (≈1 bar air). Thereafter, the first experiments on two IL systems were performed (in HV and for comparison under ambient conditions). For all PD measurements presented herein, we used a cannula with Ø_o_ = 2.02 mm and Ø_i_ = 0.5 mm (for the reasons for choosing this size, see above and SI).

### 3.1. Water

The surface tension measurements were performed with Millipore water (resistivity 18.2 MΩ·cm) at room temperature (295.45 K, literature value for γ at this temperature: 72.34 mN/m [45]) at 1 bar ambient pressure to find the optimum conditions/positions for the light source and the high-speed camera relative to the setup and to calibrate the whole system. Images were taken at different contrast and brightness settings in the SCA 22/15 software from DataPhysics (see Figure S3 SI) to illustrate the influence on the drop contour and, thus, on the resulting *γ* values using the density value for water of 0.9990 g/cm^3^ [46]. With settings for contrast of 16 and brightness of 12 (and also with slightly lower or higher brightness), the obtained surface tension values of 72.40 ± 0.06 mN/m deviate with the literature value by less than 0.1% (see Table 1 and Figure S3 in the SI). Thus, these settings were also used for all PD measurements in the following.

### 3.2. Benzene

To crosscheck our calibration, PD measurements were also carried out for benzene (C_6_H_6_) in air at room temperature (p≈ 1 bar, 295.15 K, see Table 1), using the settings deduced for water (see above). The surface tension values were determined using the density for benzene at this temperature and compared with the values from Ref. [47], obtained under equilibrium benzene vapour pressure conditions (note that we were not able to apply these conditions in our setup). Our determined surface tension of 28.85 ± 0.03 mN/m falls slightly above the literature value (28.62 mN/m at 295.15 K [48]). Note that this 0.8% deviation falls in the statistical uncertainty range of ± 2% typically observed for surface tensions values measured with the PD method [18,49]. In contrast to water, we partly attribute the slightly higher surface tension value of benzene as compared to literature to the higher benzene vapour pressure and to the non-equilibrium conditions in our setup. As can be directly observed in the videos, the formed benzene drops slowly evaporate during the image recording, which leads to a decrease in drop volume and more spherical drop shapes; thus, the prerequisite of a stable pendant drop is not fully fulfilled. Since the system is operated manually, no μL addition to the drop can be performed to compensate for the loss due to evaporation, as is often done in literature [38,50,51]. However, since our system was designed for surface tension measurements of non-volatile liquids, this evaporation issue is not relevant for our targeted measurements on ILs. Finally, we want to emphasize again the advantage of our video recording approach, which also proved very useful for the evaluation of the surface tension for liquids with a high vapour pressure, since it allows one to record the shape of the drop in any state, particularly before it becomes too small.

## 4. Pendant Drop Measurements on Ionic Liquids in High Vacuum

### 4.1. 1-Methly-3-Octylimidazolium Hexafluorophosphate [C_8_C_1_Im][PF_6_] in Vacuum and Air

**Figure 7:**
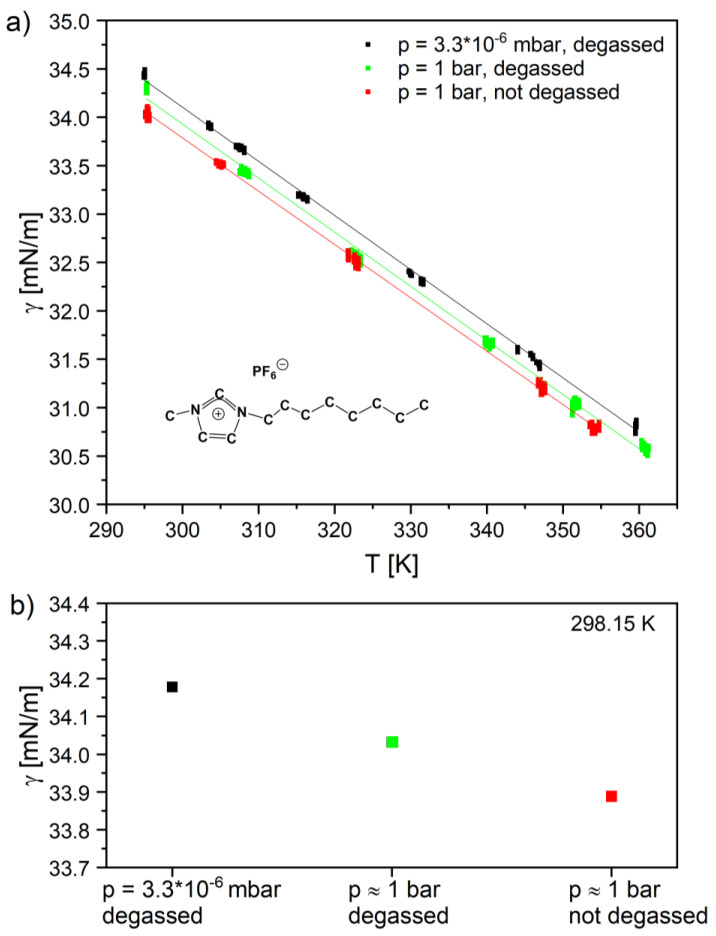
Surface tension of [C_8_C_1_Im][PF_6_] (see inset) in vacuum and in air (p ≈ 1 bar) under degassed and non-degassed conditions at different temperatures (**a**) using the temperature-dependent density of dried [C_8_C_1_Im][PF_6_] from Ref. [52] (ST values are provided in Table S1 of SI). (**b**) Calculated ST values at room temperature (298.15 K) for the three different conditions (see also Table 2) based on the linear fits of (a) using Equation (1).

As the first IL system, we investigated the surface tension of the common IL [C_8_C_1_Im][PF_6_] in a high vacuum and in air. We started with the high vacuum measurements. After inserting [C_8_C_1_Im][PF_6_] into the PD reservoir, the chamber was slowly pumped down and then heated to ≈360 K for IL degassing during 12 h at this temperature. Thereafter, we measured the surface tension at a pressure of 3.3·10^-6^ mbar from 360 down to 295 K. As a next step, we vented the chamber with air and measured the surface tension of the HV-degassed IL in the same temperature range at ambient pressure of ≈1 bar. For comparison, we also performed surface tension measurements of the initial, that is, the non-degassed [C_8_C_1_Im][PF_6_], at a pressure of ≈1 bar in a similar temperature range. For all datasets, we used the temperature-dependent density of dried [C_8_C_1_Im][PF_6_] from Ref. [52] to evaluate the surface tension; the corresponding ST data are shown in Figure 7a (individual values are provided in Table S1 of SI along their uncertainties, which are typically the size of the data points shown). In all cases, a linear temperature dependence was observed, which was fitted by the following equation:

(1) $$\gamma_{\mathrm{calc}}={\;\gamma }_{0}+{\;\gamma }_{1}\cdot T\;$$

where γ*_o_* and γ*_1_* are the fitting coefficients and *T* the temperature in Kelvin. With this equation and the corresponding coefficients, the surface tension values were calculated for the specific temperature of 298.15 K (see Table 2) and plotted for the different measurement conditions (see Figure 7b). For the degassed IL at 298.15 K in air under ambient pressure, we deduced a surface tension value of 34.03 mN/m. This value agrees to within 0.24% with the one obtained in Ref. [53] with the Wilhelmy Plate method under similar conditions (33.95 mN/m for degassed [C_8_C_1_Im][PF_6_], ambient pressure, 298.13 K, see Table 2), while it deviates by approx. 2% from the value measured by Freire et al. with the Du Noüy ring method (34.87 mN/m for degassed [C_8_C_1_Im][PF_6_], ambient pressure, 298.15 K, Ref. [54]). However, deviations of several % are quite common when comparing differences in ABSOLUTE surface tensions values from different groups [18,49]. Nevertheless, much smaller RELATIVE changes in surface tension, e.g., induced by a changed water content, can be reliably detected with high accuracy when using an identical apparatus, as was done herein.

As the next step, we measured the ST of the degassed IL in HV at 298.15 K and obtained a value of 34.18 mN/m. When comparing this value with the values for the degassed IL in air (34.03 mN/m) and for the non-degassed IL (33.89 mN/m), the surface tension was found to be systematically lower for the degassed IL at atmospheric pressure in air and lowest for the non-degassed IL in air (see Figure 7b and Table 2). Note, the observed differences were clearly larger than the reproducibility of the measurements with our apparatus of ±0.03 mN/m. We attribute these systematic differences in ST predominately to water uptake of the degassed IL after venting the chamber (the density difference between vacuum and the gas phase is expected to yield an effect of smaller than 0.1% = 0.03 mN/m) and the overall higher water content of the non-degassed IL, respectively. The water content in the IL is not known in our measurements, but the measurement method is accurate enough to detect a clear influence on the surface tension.

The influence of water on the surface tension of [C_8_C_1_Im][PF_6_] and other [PF_6_]^-^ anion-based ILs was also studied by Freire et al. [54,55]. In their ambient pressure measurements, they also found a very small decrease in surface tension of [C_8_C_1_Im][PF_6_] with increasing water content, up to 0.05 mole fraction. Moreover, the relative decrease in surface tension with increasing temperature was found to be of the same order of magnitude (−0.06 mN/m per Kelvin) [55] as ours (−0.06 mN/m per Kelvin).

### 4.2. Bis-Polyethylene Glycol-Functionalized Imidazolium Iodides [(mPEG_n_)_2_Im]I in Vacuum

**Figure 8:**
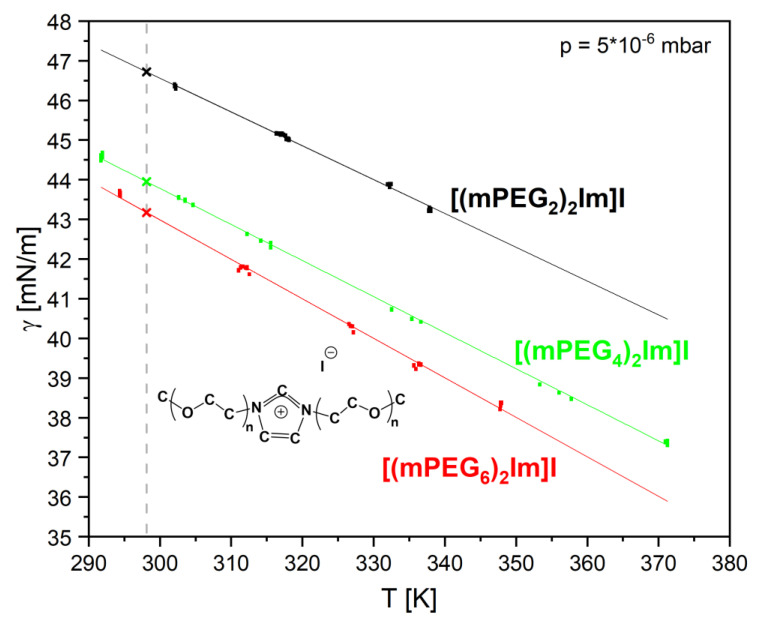
Surface tension values (see also Table S2 of SI) of the three degassed ILs [m(PEG_n_)_2_Im]I (see inset) with *n* = 2, 4, 6 measured under vacuum (p = 5·10^−6^ mbar) for different temperatures; symbol heights correspond to measurement error; crosses at 298.15 K (dashed line) represent the values given in Table 3 based on the linear fits according to Equation (1).

As a second IL example, we investigated the surface tension of three relatively new bis-polyethylene glycol-functionalized ILs [(mPEG_n_)_2_Im]I with symmetric PEG-chains at the imidazolium cation of increasing lengths (*n* = 2, 4, 6; structure, see Figure 8). These ILs were synthesized in the group of K. Meyer and very recently characterized by many methods (including density and surface tension measurements in 1 bar inert argon atmosphere with a conventional PD setup); note that IL purity as well as the absence of surface contaminations was also verified by NMR and angle-resolved XPS, respectively [56]. In this work, we focus on the surface tension data measured for the first time under HV conditions with our new setup. Prior to the measurements, each of the three ILs was carefully degassed at around 365 K for at least 17 h in HV. Thereafter, the pendant drop contours were measured as a function of temperature at a pressure of 5·10^−6^ mbar. Using the IL density values for the corresponding temperatures from Ref. [56], the derived surface tension values are plotted in Figure 8. The experimental uncertainty—which is the reproducibility given by our setup—is, in most cases, below ± 0.05 mN/m (see Table S2 of SI) and is, thus, typically about the size of the symbols. The data are fitted with a linear behaviour according to Equation (1), with corresponding fitting parameters shown in Table 3 along the surface tension values in HV at 298.15 K for the three ILs with different PEG-chain lengths (see also dashed line in Figure 8). The obtained values are in very good agreement, with surface tension values under 1 bar argon [56] measured with a completely different PD setup, with an average deviation of ± 0.5% (see Table 3). The very small deviation between the two setups is attributed to the careful calibration procedure of both instruments using water; in addition, note that drop changes due to evaporation also do not have an influence on the determined surface tension (see Figure S4 SI) as compared to the calibration measurements with benzene (see above).

With increasing temperature, we again observe a linear decrease in surface tension. When comparing the different ILs, we find that the surface tension decreases with increasing PEG_n_ chain length *n*. At 298.15 K, the surface tension decreases from 46.72 mN/m for PEG_2_ to 43.95 mN/m for PEG_4_, to 43.17 mN/m for PEG_6_ (see Table 3); that is, the surface tension decreases by –5.9% when increasing the PEG-chain length from 2 to 4 and only by –1.8% when increasing the PEG-chain length from 4 to 6. Our PD measurements in HV are accompanied by ARXPS measurements in UHV: the latter clearly indicate a preferential orientation of the PEG chains towards the surface and a depletion of the iodine anion, which increases with increasing chain length [56]. The pronounced decrease in ST from PEG_2_ to PEG_4_ and the second moderate decrease from PEG_4_ to PEG_6_ can be understood in terms of a simplified Langmuir principle, which relates surface tension to composition at the outermost surface [8]. Above a certain PEG-chain length, the surface layer is more or less fully saturated with PEG-chain constituents and, thus, the surface tension levels off. A similar behaviour has also been reported for [C_n_C_1_Im][Tf_2_N] anion-based ILs with different alkyl chain lengths [8]. Here, ARXPS measurements also revealed an orientation of the alkyl chains towards the surface, which goes along with the observation that above a certain chain length, the surface tension remains more or less constant due to saturation of the alkyl chains being present at the outer surface.

## 5. Summary and Conclusions

We described and characterized a new experimental setup for measuring the surface tension by the pendant drop method and the interface tension by the sessile drop method (contact angle) in the pressure range from 10^−7^ mbar up to 1 bar for non-volatile liquids such as ionic liquids (ILs). Such temperature-dependent measurements on well-defined ILs, as characterized by in situ angle-resolved photoelectron spectroscopy (ARXPS) under ultraclean vacuum conditions, allow for determining intrinsic surface tension values of ILs and interface tension values of ILs in contact with solid surfaces without the influence of additional surface contaminations, which are known to be omnipresent for ILs and solids. This information is instrumental for a fundamental understanding of microscopic properties, such as composition and ion orientation at the surface/interface layer, which contribute to the macroscopic surface and interface tension. Such data also serve as a benchmark for theoretical calculations and molecular dynamic simulations. Using the pendant drop method, the surface tensions of two different IL systems were determined, and for the sessile drop method, proof-of-principle experiments were performed. The pendant drop measurements at different pressures and gas ambiences provide insights into the influence of these variables on the surface tension with very high accuracy. Compared to [C_8_C_1_Im][PF_6_] measured in vacuum, we indeed observed a significant decrease in surface tension when measured in air, which is attributed to the uptake of water in the case of this IL. The temperature-dependent surface tension values of [m(PEG_n_)_2_Im]I ILs with different PEG chain lengths (*n* = 2, 4, 6) showed very good agreement with previously published data of these ILs measured under 1 bar Ar in a completely different setup. The reported decrease in surface tension with increasing chain length chains could be confirmed by our vacuum data and is attributed to an increasing enrichment of the PEG chains at the surface found in ARXPS.

## 6. Materials

Surface tension measurements were performed with Millipore water (resistivity 18.2 MΩ·cm), analytically benzene (C_6_H_6_) (purity 99.7%) purchased from Merck, acquired from Sigma-Aldrich and 1-Methly-3-octylimidazolium hexafluorophosphate [C_8_C_1_Im][PF_6_] (purity 99%), purchased from IoLiTec and used as delivered. The [m(PEG_n_)_2_Im]I with (*n* = 2, 4, 6) were synthesized by V. Seidl according to Ref. [56].

## Figures and Tables

**Table 1:** Surface tension reference measurements with water and benzene at room temperature and atmospheric pressure (p ≈ 1 bar). Density and literature surface tension for water are from Refs. [45,46] and for benzene from Refs. [47,48]. The maximum error in the surface tension compared to the literature values is 0.1% for water and 0.8% for benzene.

Reference Liquid	*T* (K)	*ρ* (g/cm^3^)	*γ* (mN/m)	*γ* (mN/m) Literature	Standard Deviaton Δ*γ* (mN/m)	Maximum Deviaton $\Delta \gamma =\frac{\left|\gamma_{min}-\gamma_{max}\right|}{2}$ (mN/m)	Deviation from Literature Value (%)
**MP-water**	295.45	0.9990 [46]	72.40 ± 0.06	72.34 [45]	0.06		<0.1
**Benzene**	295.15	0.8767 [47]	28.85 ± 0.03	28.62 [48]	0.23	0.03	0.8

**Table 2:** Surface tension of [C_8_C_1_Im][PF_6_] at different pressures under degassed and non-degassed conditions (different water content) at 298.15 K. The surface tension values are calculated using the temperature-dependent density of dried [C_8_C_1_Im][PF_6_] from Ref. [52] (1.2383 g/cm^3^ at 298.15 K). Coefficients $\gamma_{0}$ and $\gamma_{1}$ of Equation (1) are also given, along the R^2^ values of the linear fits.

Condition	*p* (mbar)	*γ* (mN/m) (T = 298.15 K)	*γ* (mN/m) from Ref [53] (T = 298.13 K)	*γ*_0_ (mN/m)	*γ*_1_ (mN/m·K^−1^)	*R* ^2^
Degassed	3.3·10^−6^	34.18		50.88	–0.0559	0.999
Degassed	1.0·10^3^	34.03	33.95	50.69	–0.0559	0.997
Non-degassed	1.0·10^3^	33.89		50.32	–0.0551	0.999

**Table 3:** Surface tension values of [(mPEG_n_)_2_Im]I with *n* = 2, 4, 6 at T = 298.15 K measured under vacuum in comparison to literature data measured under 1 bar Ar atmosphere [56]. Coefficients $\gamma_{0}$ and $\gamma_{1}$ of Equation (1) for the temperature-dependent surface tension are also given along the R^2^ values of the linear fits.

IL	*γ* (mN/m) at 5·10^−6^ mbar (T = 298.15 K)	*γ* (mN/m) from Ref. [56] at 1 bar Ar (T = 298.15 K)	*γ*_0_ (mN/m)	*γ*_1_ (mN/m·K^−1^)	*R* ^2^
[(mPEG_2_)_2_Im]I	46.72	46.26	72.17	–0.0854	0.997
[(mPEG_4_)_2_Im]I	43.95	43.94	71.05	–0.0909	0.999
[(mPEG_6_)_2_Im]I	43.17	42.96	72.87	–0.0996	0.997

## Supplementary Materials

The following supporting information can be downloaded at: https://www.mdpi.com/article/10.3390/ijms232113158/s1. References [57,58,59,60,61].
