# Supplementary material for: Probing Surface and Interfacial Tension of Ionic Liquids in Vacuum with the Pendant Drop and Sessile Drop Method
Source: Int J Mol Sci. 2022 Oct 29;23(21):13158. doi: 10.3390/ijms232113158 (PMC9658333; doi:10.3390/ijms232113158)
Supplement: Supplementary file 1 [file ijms-23-13158-s001.zip › IJMS-Adv-ILs_Paap-et-al_SI.pdf]

Supporting Information

Probing Surface and Interfacial Tension of Ionic Liquids in Vacuum with the Pendant Drop and Sessile Drop Method

Ulrike Paap[1] *ORCID: 0000-0002-1442-0239*, Bernd Kreß[1], Hans-Peter Steinrück[1] *ORCID: 0000-0003-1347-8962*, Florian Maier[1]* *ORCID: 0000-0001-9725-8961*

Optimizing cannula dimensions

In addition to the setup calibration in air using ultrapure water (see main text), PD measurements were also performed in air using the ionic liquid (IL) 1-Methly-3-octylimidazolium hexafluorophosphate ($[C_8C_1Im][PF_6]$) at different inner and outer cannula diameters. Here, systematic changes in the surface tension were detected: the derived surface tension (ST) values are largest with smallest outer cannula diameter, slightly decreases with increasing outer diameter and starts to increase again for a very large outer cannula diameter. The reason for this behavior is attributed to the difference in drop shape for different cannula diameters [1]. Very small cannulas form very small drops with practically no deformation due to gravity and thus, the drops are rather round [2-4]. For spherical drops, the bond number is very small and the error in ST grows as the bond number decreases [5]. This leads to a greater difference in the individual ST values for each measured drop. On the other end, diameters that are very large can form droplets with a large volume, but the constriction at the neck of the droplet is also not very pronounced [6,7] as for the very small cannulas. Since the constriction at the drop neck is a decisive factor for reliable surface tension determination, we decided to use a cannula with an outer diameter of $Ø_o$ = 2.02 mm and $Ø_i$ = 0.5 mm made of stainless-steel (note that the inner diameter does not influence the surface tension if the wettability of the cannula is given [8]; cannula diameters of at least 1.65 mm for PD are also recommended by different manufacturers such as Krüss and DataPhysics).

Drop stability in HV

In contrast to the situation under atmospheric pressure, several difficulties occurred before stable pendant drops of an ionic liquid in vacuum could be measured. Starting with conventional PD cannula dimensions with rather large inner diameters at the beginning, the emerging drops quickly fell off due to the excess of gravitational force without the counterbalancing atmospheric pressure. The only forces that counteract gravity and keep the drop at the tip's end are capillary forces. We tested stainless-steel cannula sizes with different inner and outer diameters. The smaller the inner diameter compared to the outer diameter of the cannula, the more stable the drop was. However, 0.5 mm as inner bore was the minimum limit for practical drilling reasons, which was not sufficient to form stable IL drops. We thus increased the capillary forces by simply inserting two twisted stainless-steel wires (0.2 mm in diameter) into the 0.5 mm cannula bore to further reduce the inner diameter and increase contact surface and thereby the capillary forces. By using this approach, stable drops were successfully formed. For all HV measurements presented here, the twisted wires were always present to ensure stability of the drops even at higher temperatures (see Fig. S2).

Bubble formation in HV

Another very serious problem turned out to be gas bubble formation during the drop formation in HV. Correct degassing of the ILs was thus crucial. Otherwise, bubbles regularly appeared during drop formation, which then caused drop shape deformations and additional IL wetting problems at the outer cannula after bubble bursts accompanied by IL spilling inside the vacuum chamber (see also video SI-movie1-bubble-formation-live.mp4, SI). For the degassing of the ILs measured so far, a reservoir temperature of at least 70 °C to 100 °C was necessary depending on IL with a degassing time of about 8 to 10 hours while applying a vacuum better than 10^{-5} mbar.

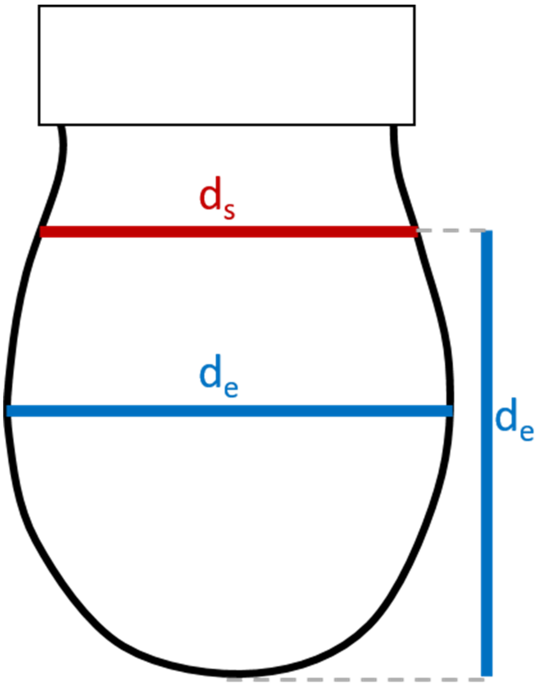

Figure S1: Representation of the "Method of selected planes" from Ref. [9] using the ratio $S = d_s/d_e$ as characteristic descriptor for the pendant drop shape, with the maximum drop diameter d_e (blue) and the drop diameter d_s (red) at the distance d_e above the apex.

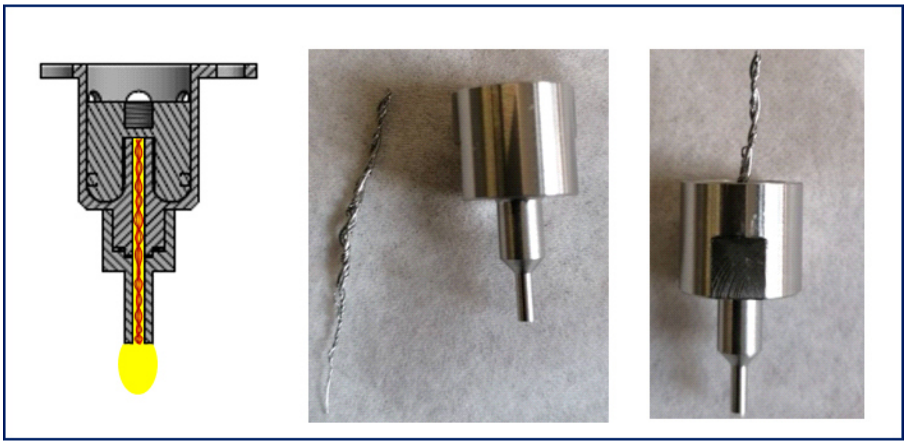

Figure S2: Left: Schematic representation of the twisted wires in the cannula, middle: Photo of the twisted wires and the cannula and right: Photo of the twisted wires inserted into the cannula.

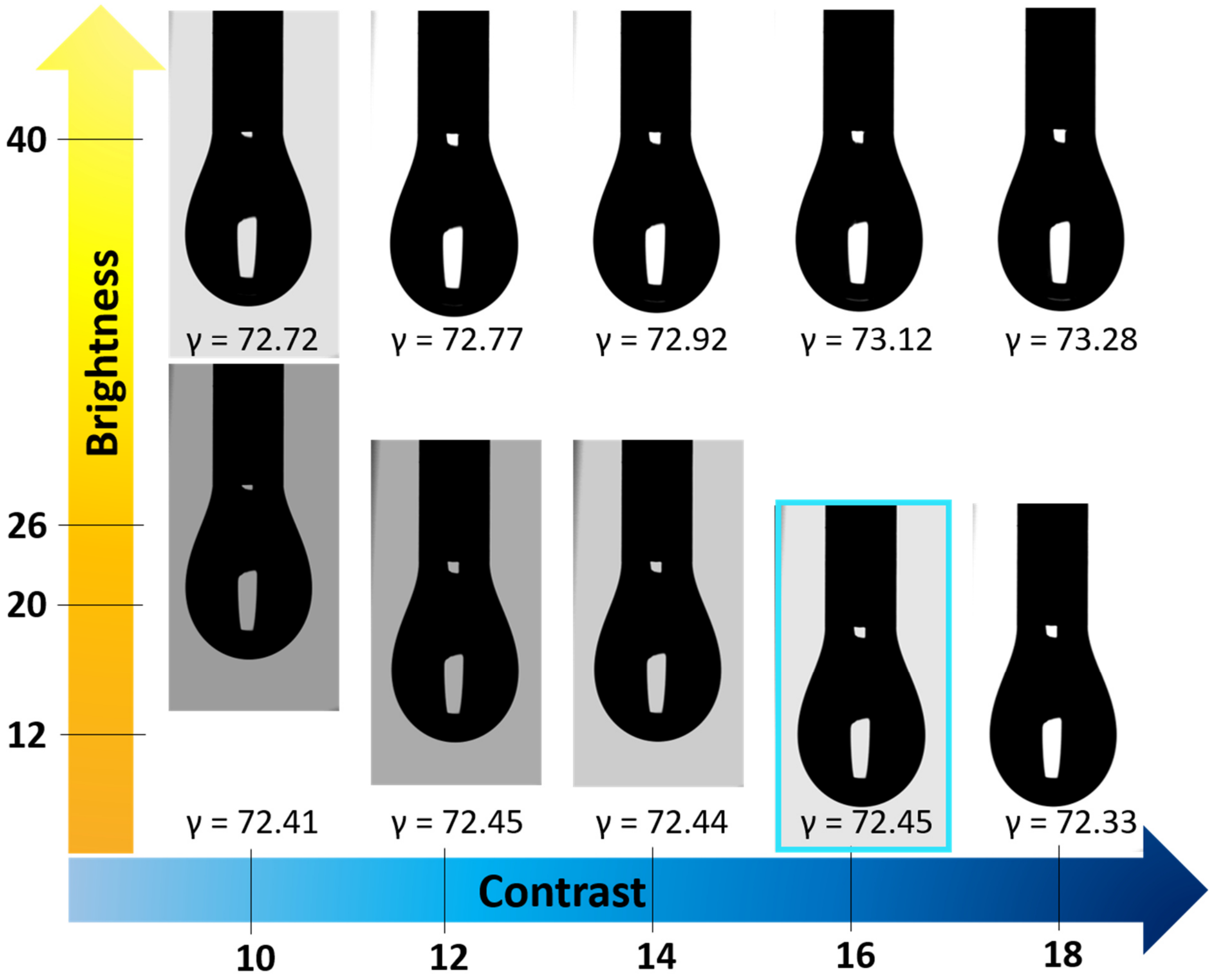

Figure S3: *Photographs of Millipore water taken at different contrast and brightness settings in the software SCA 22/15. The drop with the final settings for this work (brightness 12, contrast 16) is outlined in turquoise.*

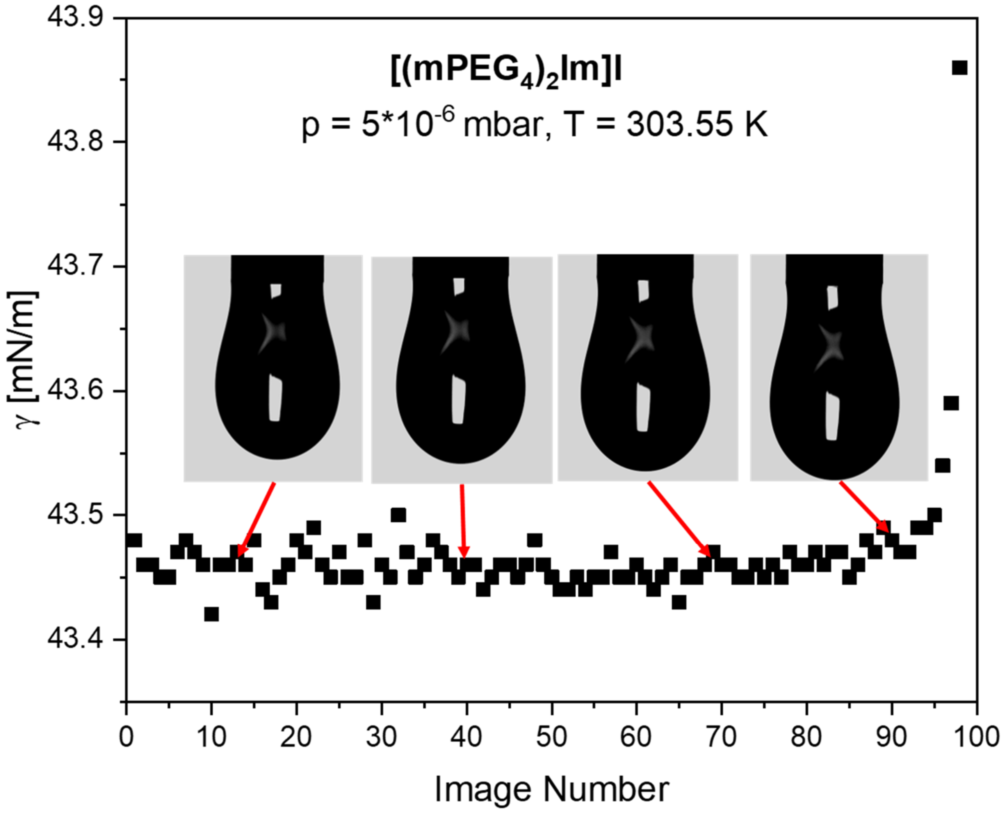

__Figure S4__: Surface tension of [(mPEG$_4$)$_2$Im]I at T = 303.55 K derived from a 20 second video (rate of 5 fps); drop fell off after image 100 (see also time lapse video SI-movie2-IL-drop-FigS4-5xfaster.mp4, SI).

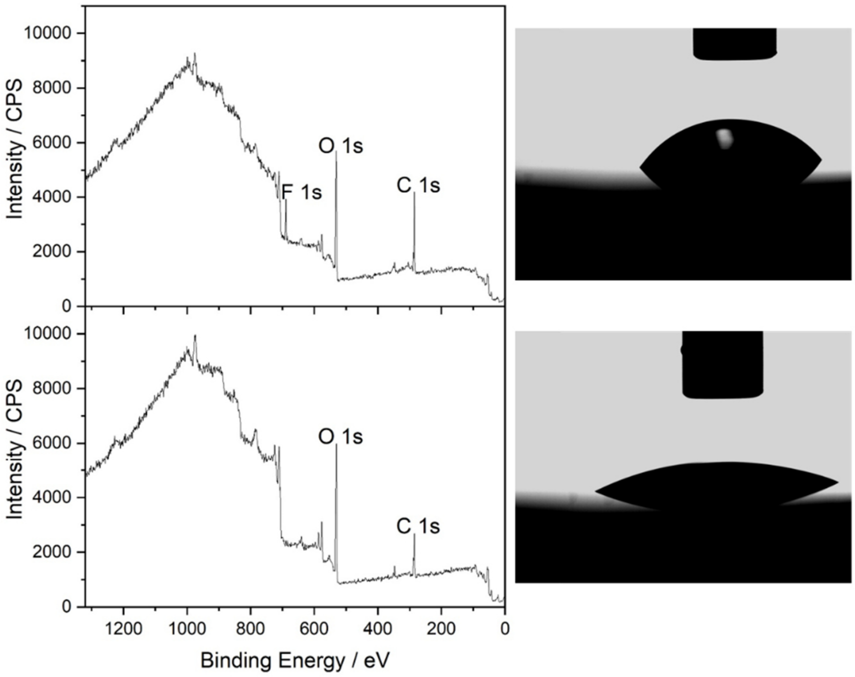

CA left (°)	53.6
CA right (°)	53.8
CA mean value (°)	53.7

CA left (°)	23.5
CA right (°)	23.5
CA mean value (°)	23.5

***Figure S5**: Comparison of room temperature contact angles and thus, wetting behavior, of [C_8C_1Im][PF_6] on stainless-steel supports with different levels of carbon and fluorine contamination. Left: XPS surveys of the supports measured in 0° in UHV, middle: images of the drops on the corresponding surfaces recorded in air, right: related contact angles (CA) of the corresponding images.*

***Table S1**: Individual surface tension values γ plotted in Fig. 7a of [C_8C_1Im][PF_6] at different pressures under degassed and non-degassed conditions and at different temperatures. At a given temperature, each γ value is obtained by averaging over the ST values derived from several pictures of at least two stable pendant drops (maximum deviations $\Delta_{max}\gamma$ of the corresponding average values γ are also given).*

[C_8C_1Im][PF_6]								
Degassed (p = $3.3\cdot10^{-6}$ mbar)			Degassed (p = $1.0\cdot10^{3}$ mbar)			Non-degassed (p = $1.0\cdot10^{3}$ mbar)		
T [K]	γ [mN/m]	$\Delta_{max}\gamma$ [mN/m]	T [K]	γ [mN/m]	$\Delta_{max}\gamma$ [mN/m]	T [K]	γ [mN/m]	$\Delta_{max}\gamma$ [mN/m]
294.95	34.43	±0.02	295.25	34.29	±0.04	295.15	34.03	±0.03
295.05	34.44	±0.05	295.35	34.31	±0.06	295.35	34.04	±0.08
303.45	33.91	±0.03	307.55	33.44	±0.02	295.45	34.10	±0.01
303.65	33.90	±0.02	307.75	33.46	±0.03	295.55	34.06	±0.03
303.75	33.90	±0.02	307.95	33.44	±0.02	295.65	34.00	±0.04
307.35	33.70	±0.02	308.75	33.40	±0.02	304.45	33.53	±0.02
307.55	33.68	±0.02	321.95	32.60	±0.02	304.75	33.50	±0.01
307.75	33.68	±0.02	322.25	32.60	±0.09	304.85	33.51	±0.01
307.95	33.67	±0.01	322.95	32.57	±0.07	304.95	33.53	±0.02
308.15	33.66	±0.03	323.25	32.55	±0.05	305.15	33.49	±0.01
315.25	33.20	±0.02	323.45	32.53	±0.06	305.35	33.52	±0.02
315.85	33.18	±0.02	339.75	31.70	±0.05	305.45	33.51	±0.01
315.95	33.17	±0.02	339.95	31.68	±0.05	321.75	32.58	±0.05
316.35	33.16	±0.01	340.35	31.65	±0.05	321.85	32.56	±0.04
316.45	33.15	±0.02	340.75	31.66	±0.04	322.55	32.54	±0.04
329.75	32.40	±0.01	351.25	31.00	±0.07	322.65	32.50	±0.06
329.95	32.38	±0.02	351.35	31.02	±0.04	322.85	32.52	±0.04
330.15	32.37	±0.01	351.45	31.06	±0.05	323.15	32.50	±0.07
331.35	32.30	±0.03	351.65	31.05	±0.05	346.75	32.25	±0.04
331.45	32.32	±0.00	351.95	31.05	±0.03	347.05	31.27	±0.02
331.65	32.30	±0.03	352.05	31.03	±0.03	347.15	31.19	±0.06
344.05	31.60	±0.03	352.15	31.04	±0.03	347.25	31.15	±0.02
345.75	31.55	±0.01	360.35	30.63	±0.03	347.55	31.21	±0.04
346.05	31.53	±0.03	360.45	30.59	±0.03	347.65	31.19	±0.05
346.55	31.46	±0.01	360.65	30.60	±0.03	353.55	30.81	±0.07
346.85	31.45	±0.02	360.85	30.58	±0.04	353.95	30.77	±0.03
346.95	31.44	±0.04	361.15	30.56	±0.06	354.25	30.77	±0.04
359.55	30.77	±0.06	361.25	30.58	±0.02	354.45	30.78	±0.02
359.65	30.84	±0.04				354.75	30.82	±0.06

***Table S2**: Individual surface tension values γ plotted in Fig. 8 for the three degassed ILs $[m(PEG_n)_2Im]I$ (n = 2, 4, 6) measured in vacuum (p = 5*10^{-6} mbar) at different temperatures (values obtained by averaging over several drops and pictures at a given temperature; maximum deviations $\Delta_{max}\gamma$ of the corresponding average values γ are also given).*

$[(mPEG_2)_2Im]I$			$[(mPEG_4)_2Im]I$			$[(mPEG_6)_2Im]I$		
T [K]	γ [mN/m]	$\Delta_{max}\gamma$ [mN/m]	T [K]	γ [mN/m]	$\Delta_{max}\gamma$ [mN/m]	T [K]	γ [mN/m]	$\Delta_{max}\gamma$ [mN/m]
302.05	46.37	±0.03	291.75	44.52	±0.10	394.35	43.65	±0.07
302.25	46.31	±0.06	291.95	44.62	±0.06	394.45	43.63	±0.05
316.35	45.17	±0.02	302.65	44.55	±0.02	311.05	41.71	±0.02
316.75	45.16	±0.02	303.55	43.47	±0.03	311.35	41.79	±0.02
316.95	45.13	±0.01	304.65	43.37	±0.02	311.65	41.81	±0.01
317.15	45.14	±0.03	312.25	42.63	±0.01	312.05	41.78	±0.01
317.35	45.12	±0.02	314.15	42.46	±0.01	312.25	41.78	±0.02
317.65	45.10	±0.02	315.55	42.31	±0.03	312.55	41.61	±0.01
317.75	45.03	±0.02	332.55	40.72	±0.02	326.25	39.70	±0.02
317.95	45.03	±0.03	335.35	40.49	±0.01	326.55	40.36	±0.01
318.05	45.03	±0.02	336.65	40.41	±0.01	326.75	40.30	±0.02
318.15	45.01	±0.02	353.35	38.84	±0.01	327.05	40.30	±0.01
331.95	43.88	±0.01	356.05	38.63	±0.01	327.15	40.15	±0.01
332.25	43.84	±0.06	357.75	38.47	±0.01	335.65	39.32	±0.02
332.45	43.89	±0.01	371.15	37.40	±0.02	335.95	39.23	±0.01
337.25	43.22	±0.02	370.95	37.39	±0.02	336.35	39.34	±0.03
337.85	43.28	±0.02	371.25	37.37	±0.03	336.65	39.33	±0.02
337.95	43.26	±0.01				347.75	38.21	±0.01
338.05	43.23	±0.03				347.85	38.34	±0.05
						347.95	38.37	±0.02

References:

1. Lin, S.-Y.; Hwang, H.-F. Measurement of low interfacial tension by pendant drop digitization. *Langmuir* **1994**, *10*, 4703-4709, doi:10.1021/la00024a052.
2. Berry, J.D.; Neeson, M.J.; Dagastine, R.R.; Chan, D.Y.C.; Tabor, R.F. Measurement of surface and interfacial tension using pendant drop tensiometry. *J. Colloid Interface Sci.* **2015**, *454*, 226-237, doi:10.1016/j.jcis.2015.05.012.
3. Hoorfar, M.; W. Neumann, A. Recent progress in Axisymmetric Drop Shape Analysis (ADSA). *Adv. Colloid Interface Sci.* **2006**, *121*, 25-49, doi:10.1016/j.cis.2006.06.001.
4. Morita, A.; Carastan, D.; Demarquette, N. Influence of drop volume on surface tension evaluated using the pendant drop method. *Colloid Polym. Sci.* **2002**, *280*, 857-864, doi:10.1007/s00396-002-0694-z.
5. Alvarez, N.J.; Walker, L.M.; Anna, S.L. A non-gradient based algorithm for the determination of surface tension from a pendant drop: Application to low Bond number drop shapes. *J. Colloid Interface Sci.* **2009**, *333*, 557-562, doi:10.1016/j.jcis.2009.01.074.
6. Yang, J.; Yu, K.; Zuo, Y.Y. Accuracy of Axisymmetric Drop Shape Analysis in Determining Surface and Interfacial Tensions. *Langmuir* **2017**, *33*, 8914-8923, doi:10.1021/acs.langmuir.7b01778.
7. Hoorfar, M.; Neumann, A.W. Axsymmetric drop shape analysis (ADSA) for the determination of surface tension and contact angle of water. *J. Adhes.* **2004**, *80*, 727-743, doi:10.1080/00218460490477684.
8. Hoorfar, M.; Kurz, M.A.; Neumann, A.W. Evaluation of the surface tension measurement of axisymmetric drop shape analysis (ADSA) using a shape parameter. *Colloids Surf. A Physicochem. Eng. Asp.* **2005**, *260*, 277-285, doi:10.1016/j.colsurfa.2004.08.080.
9. Andreas, J.M.; Hauser, E.A.; Tucker, W.B. Boundary tension by pendant drops 1. *J. Phys. Chem.* **1938**, *42*, 1001-1019, doi:10.1021/j100903a002.
